# Comparative Proteome Research in a Zebrafish Model for Vanishing White Matter Disease

**DOI:** 10.3390/ijms22052707

**Published:** 2021-03-08

**Authors:** Doeun Kim, Yu-Ri Lee, Tae-Ik Choi, Se-Hee Kim, Hoon-Chul Kang, Cheol-Hee Kim, Sangkyu Lee

**Affiliations:** 1 BK21 FOUR Community-Based Intelligent Novel Drug Discovery Education Unit, College of Pharmacy and Research Institute of Pharmaceutical Sciences, Kyungpook National University, Daegu 41566, Korea; kdkdl1230@gmail.com; 2 Department of Biology, Chungnam National University, Daejeon 34134, Korea; jhyrsanta@naver.com (Y.-R.L.); c860523@naver.com (T.-I.C.); 3 Division of Pediatric Neurology, Department of Pediatrics, Epilepsy Research Institute, Pediatric Epilepsy Clinic, Epilepsy Research Institute, Severance Children’s Hospital, Yonsei University College of Medicine, Seoul 03772, Korea; SEHEEKIM@yuhs.ac (S.-H.K.); HIPO0207@yuhs.ac (H.-C.K.)

**Keywords:** Vanishing White Matter disease, EIF2B, comparative proteomics, SLC1A4

## Abstract

Vanishing white matter (VWM) disease is a genetic leukodystrophy leading to severe neurological disease and early death. VWM is caused by bi-allelic mutations in any of the five genes encoding the subunits of the eukaryotic translation factor 2B (EIF2B). Previous studies have attempted to investigate the molecular mechanism of VWN by constructing models for each subunit of EIF2B that causes VWM disease. The underlying molecular mechanisms of the way in which mutations in EIF2B3 result in VWM are largely unknown. Based on our recent results, we generated an *eif2b3* knockout (*eif2b3^−/−^*) zebrafish model and performed quantitative proteomic analysis between the wild-type (WT) and *eif2b3^−/−^* zebrafish, and identified 25 differentially expressed proteins. Four proteins were significantly upregulated, and 21 proteins were significantly downregulated in *eif2b3^−/−^* zebrafish compared to WT. Lon protease and the neutral amino acid transporter SLC1A4 were significantly increased in *eif2b3^−/−^* zebrafish, and crystallin proteins were significantly decreased. The differential expression of proteins was confirmed by the evaluation of mRNA levels in *eif2b3^−/−^* zebrafish, using whole-mount in situ hybridization analysis. This study identified proteins which candidates as key regulators of the progression of VWN disease, using quantitative proteomic analysis in the first EIF2B3 animal model of VWN disease.

## 1. Introduction

Childhood Ataxia with Central Nervous System Hypomyelination, also called Vanishing White Matter (VWM) disease, is one of the most prevalent inherited childhood leukoencephalopathies. Infantile VWM is a grave disease, and 50% of patients die within two years of diagnosis. It is a fatal, stress-sensitive leukodystrophy that mainly affects children, and for which there is currently no treatment [1]. VWM is characterized by ataxia, spasticity, and variable optic atrophy [2]. Over the past 20 years, various phenotypes of the disease have been identified, and it has been established that it can appear at all ages, not just in childhood [2,3]. VWM is a neurological disease caused by mutations in the eukaryotic translation initiation factor 2B (*EIF2B*) that produce bi-allelic pathogenic variants in one of five genes: *EIF2B1, EIF2B2, EIF2B3, EIF2B4,* or *EIF2B5* [4].

*EIF2B* is indispensable for the initiation of mRNA translation and regulation of protein synthesis under different conditions, including cell stress [1,5]. Protein synthesis, an important component of which is protein synthesis and balancing of biological processes, is a key step in the expression of a cell’s genetic information. *EIF2B* controls the first major stage of mRNA translation, initiation, which involves the recruitment of the ribosome to the mRNA and the identification of the correct start codon to commence translation. The molecular mechanisms controlling the pathological symptoms of VWM can be investigated by identifying changes in protein expression caused by mutations in *EIF2B*. In some previous studies, proteomics approaches were used to investigate the molecular mechanisms affecting VWM [6,7,8]. Although previous studies have identified mitochondrial dysfunction due to altered proteasomal activity, and impaired balance between protein synthesis and degradation, these results arise from limited investigations of effects at the cellular level, that are not related to the pathogenesis of VWM.

To understand the pathology of VWM, experimental models have been generated. In 2010, the first animal model was developed in mice, by introducing a point mutation into the mouse *Eif2b5* gene locus A [9]. However, mouse knockouts of the *Eif2b* genes were lethal, precluding their use for further study of VWM pathogenicity. Recently, an in vitro model was constructed by differentiating gray and white matter astrocytes obtained from humans and mice into induced pluripotent stem (iPS) cells, as a model for differentiation, after the transformation of patient-derived fibroblast into iPS cell was achieved [10,11]. However, the cell model cannot be applied to the phenotyping of individuals, due to the mutation, and only limited molecular changes can be studied.

Recently a model for, *EIF2B5* in VWM disease was reproduced by the truncation of *eif2b5* in zebrafish (*Danio rerio*), which produced impaired motor function, and led to further activation of the cellular integrated stress response [12]. We established the first *eif2b3* mutant animal model for VWM disease that is useful for functional validation of human gene variants, including a novel variant identified in a Korean VWM patient [13]. Following defects in the expression of myelin genes and glial cell differentiation, we observed phenotypes resembling those of human patients in *eif2b3* knockout (*eif2b3^−/−^*) zebrafish. Using the first EIF2B animal model, we used comparative quantitative proteomic analysis between WT and *eif2b3^−/−^* to understand the molecular mechanisms affecting VWM. Since the zebrafish has high genetic similarity to humans, the experimental results obtained in this study were applied to human data to identify the molecular mechanism of VWM.

## 2. Results

### 2.1. Establishment of a Vanishing White Matter Disease Model by eif2b3 Knockout

**Figure 1:**
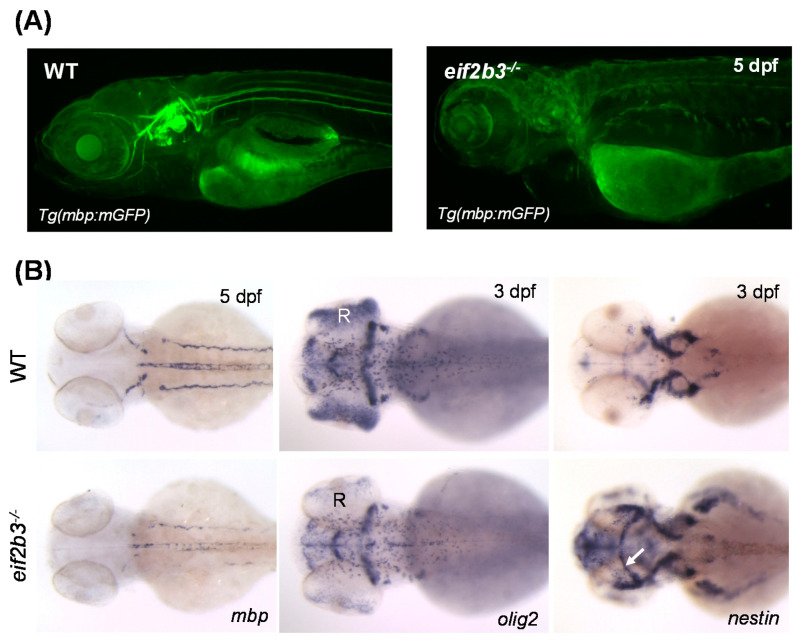
Zebrafish *eif2b3* knockout model for VWM disease. (**A**) Visualization of myelination using *Tg (mbp:mGFP)* crossed with *eif2b3^−/−^* zebrafish at 5 dpf. Myelination defects in *eif2b3^−/−^* which recapitulate phenotypes of VWM disease. (**B**) Whole-mount in situ hybridization with myelin development markers, *mbp*, *olig2*, and *nestin* in *eif2b3^−/−^* zebrafish. *mbp*, myelin basic protein; *oilg2*, oligodendrocyte transcription factor; *nestin*, early neural stem/progenitor cell marker. Arrow indicates ectopic expression of *nestin* in the midbrain region. R, retina.

In our previous study, an *eif2b3^−/−^* model was generated in zebrafish based on the identification of a new variant in Korean patients. The suitability of the model was verified by confirming the phenotype, which was typical of that associated with VWM disease. The characteristic defects in the nervous system of the *eif2b3^−/−^* zebrafish were confirmed by visualization of myelination using in transgenic zebrafish, *Tg(mbp:mGFP)* (Figure 1A). A reduction of the expression of *mbp* was confirmed by whole-mount in situ hybridization (WISH) (Figure 1B). We also investigated the altered differentiation of cell fates in glial cell progenitors in *eif2b3^−/−^* zebrafish corresponding to VWM pathogenesis. While the expression of the mature glial cell marker *olig2* was downregulated, the earlier neural cell marker *nestin* was upregulated (Figure 1B).

### 2.2. Comparative Proteome Analysis of eif2b3^−/−^ Compared to Wild-Type

**Figure 2:**
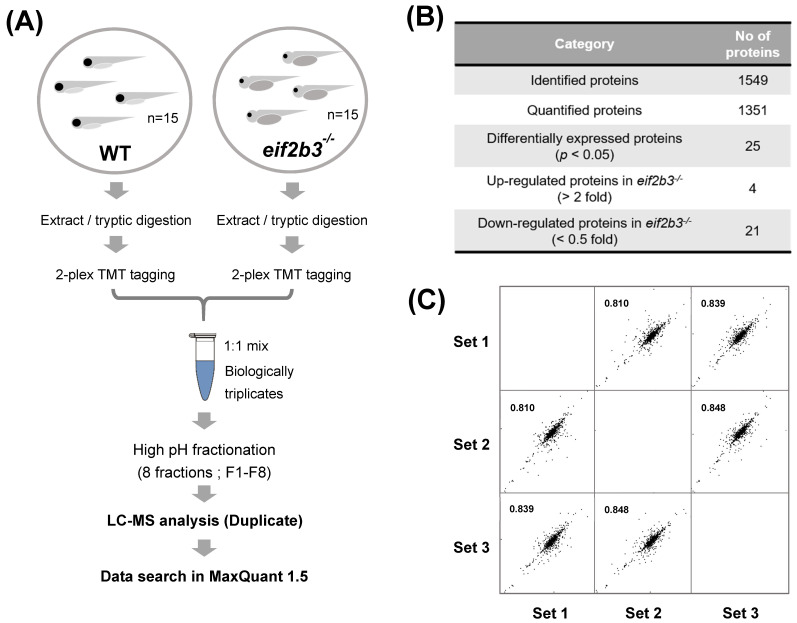
Workflow for comparative proteome analysis in an *eif2b3* knockout. (**A**) Flowchart illustrating the experimental procedure for quantification of regulated proteins in the *eif2b3^−/−^* model. (**B**) The table showing the number of proteins identified in WT and *eif2b3^−/−^* model, respectively. (**C**) Highly correlated results of triplicate analyses.

Prior to comparative proteomic analysis, yolk was removed to prevent interference in our analysis. Deyolked embryos were confirmed by sodium dodecyl sulfate-polyacrylamide gel electrophoresis (SDS-PAGE) gel staining after the removal of the yolk proteins, which showed a specific protein band at a molecular weight of 80–100 kDa (Figure S1) [14]. The protein expression between WT and *eif2b3^−/−^* zebrafish larvae were quantified using mass spectrometry-based proteomics (Figure 2A). Using the UniProt zebrafish database, we identified a total of 1549 proteins with at least two peptides in the combined WT and *eif2b3^−/−^* larvae, among which 1351 proteins were quantified based on comparative proteomic analysis (Figure 2B). The total identified protein list is presented in Supplementary Table S1. Quantitative data were collected from triplicates of prepared samples, the reproducibility of which was validated by scatter plots, with protein intensity and Pearson correlation score showing values up to 0.9 (Figure 2C).

### 2.3. Identification of Differentially Expressed Proteins in eif2b3^−/−^ Zebrafish

**Figure 3:**
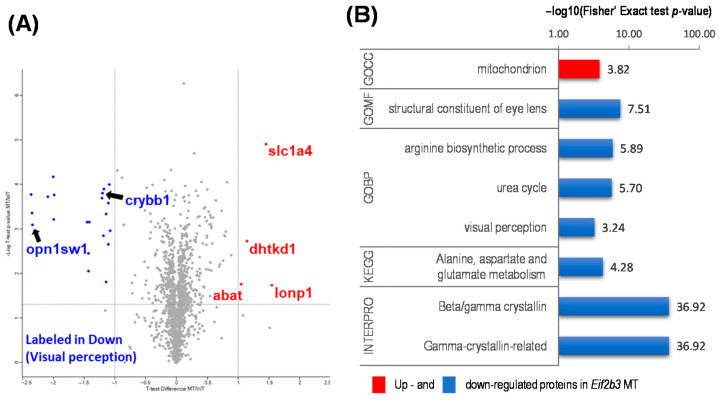
Functional annotation of differentially expressed proteins (DEP) in the WT and *eif2b3* knockout. (**A**) Distribution of the 1352 quantified proteins (upregulated, red filled circles; downregulated, blue filled circles) in *eif2b3^−/−^* compared to the WT, according to one-sample t-tests and fold change (*p* < 0.05). (**B**) DAVID-generated GO enrichment and KEGG pathway analysis of DEPs. The–log of Fisher’s exact test was used to represent the enrichment index. GOBP: Gene Ontology Biological Process, CC: cellular component, MF: molecular function, KEGG: Kyoto Encyclopedia of Genes and Genomes.

Among the proteins measured, were 25 differentially expressed proteins (DEPs) (Figure 2B). To identify significant DEPs, we used a fold-change cut-off of 2 for upregulated proteins and 0.5 for downregulated proteins, and *p* value of less than 0.05 in a volcano plot using a one-way t-test (Figure 3A). Four proteins were significantly upregulated, and 21 proteins were significantly downregulated in *eif2b3^−/−^* larvae compared to WT (Table 1). Four proteins were increased in *eif2b3^−/−^*. Three were mitochondrial proteins: Lon protease, 2-oxoglutarate dehydrogenase E1 component, and 4-aminobutyrate aminotransferase, all three of which were confirmed to be homologous with human proteins. Neutral amino acid transporter SLC1A4, encoding ASCT1, was also significantly increased in the *eif2b3^−/−^* model. In the functional annotation using Gene Ontology (GO) terms, three of the upregulated proteins were associated with mitochondria in the GO cellular component (GO CC) category (Figure 3B). According to previous studies, the functional change of mitochondria in VWM disease observed in the *eif2b* mutant is known, so the three proteins observed in this study may be related to the functional changes in mitochondria in VWM disease [15].

The direct effect of the *eif2b3* mutant which is related to VWM disease pathology can be predicted through the analysis of proteins decreased in DEP. To acquire an in-depth understanding of the biological effect of *eif2b3^−/−^*, we used GO terms, Kyoto Encyclopedia of Genes and Genomes (KEGG) pathways, and Interpro enrichment analyses (Figure 3B). The downregulated proteins in *eif2b3^−/−^* were largely divided into two groups: those of visual and those of metabolic relevance. In the eye, downregulated proteins were involved in the structure of the lens and visual perception, according to GO molecular function (GO MF) and visual perception in the GO biological process (GO BP) categories. In Interpro, 16 downregulated proteins in *eif2b3^−/−^* were annotated as Beta/gamma crystallin and Gamma-crystallin-related, which corresponds to 76.2% of the downregulated proteins. Most of the downregulated proteins were crystallin-related proteins.

Other proteins in the downregulated group in *eif2b3^−/−^* were associated with metabolism. The downregulated proteins were annotated as participating in the arginine biosynthetic process and the urea cycle in the GO BP category, and alanine, aspartate and glutamate metabolism in the KEGG pathways (Figure 3B). Citrulline--aspartate ligase and aspartate carbamoyl transferase were included among 21 downregulated proteins.

### 2.4. Validation of Differentially Expressed Proteins Using Whole-Mount In Situ Hybridization

**Figure 4:**
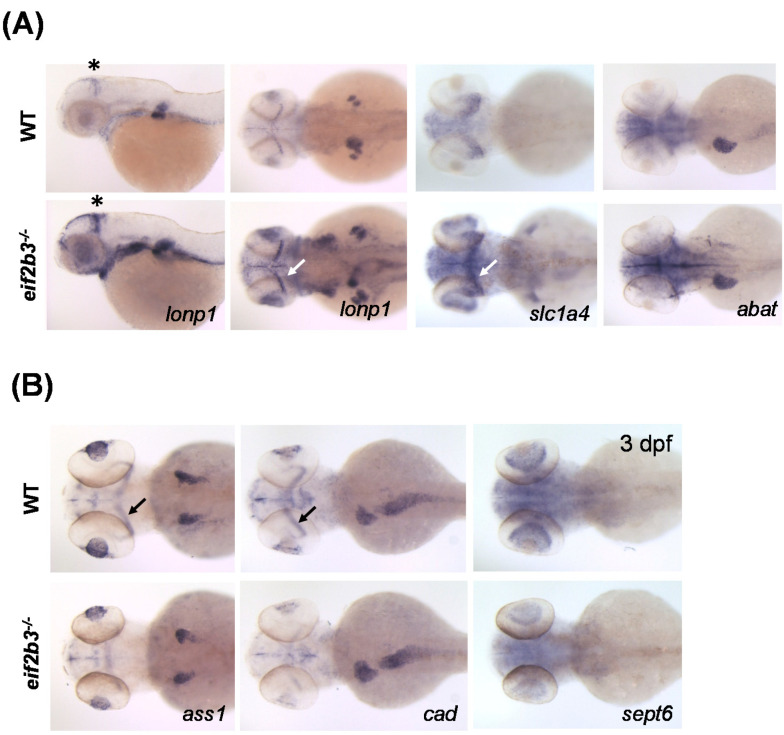
Visualization of differently expressed proteins in WT and *eif2b3* knockout zebrafish. (**A**) Whole-mount in situ hybridization of WT and *eif2b3^−/−^* probed for *lonp1*, *slc1a4*, and *abat* upregulated genes. (**B**) Whole-mount in situ hybridization of WT and *eif2b3^−/−^* probed for *ass1*, *cad*, and *sept6* downregulated genes. * The asterisk and arrow indicate the midbrain-hindbrain boundary (MHB).

In order to confirm the alteration of protein expression identified by comparative proteome analysis in *eif2b3^−/−^* compared to WT zebrafish, the mRNA expression of genes related to DEPs was performed using whole-mount in situ hybridization (WISH) analysis. The gene related to four significantly upregulated proteins is shown in Figure 4A. The mRNAs of *lonp1*, *slc1a4* and *abat* were increased in *eif2b3^−/−^*. However, the expression of *dhtkd1* was not significantly changed in the *eif2b3* mutant (Figure S2A). The mRNA expression of the DEPs increased by at least 1.5-fold in quantified proteins, and four DEPs increased by two-fold compared with WT zebrafish (Table S1). The mRNA expression of *psat1* and *xbp1* was upregulated in *eif2b3^−/−^*. The mRNA expression of the unfolded protein response pathway genes *xbp1*, *atf4*, and *atf6* were upregulated in *eif2b3^−/−^* zebrafish, as early as 2 dpf, preceding those of *slc1a4*, *lonp1*, and *psat1*, especially in the midbrain-hindbrain boundary (MHB) (Figures S2B,C and S3). The mRNA expression of downregulated proteins was also confirmed. The mRNA expression of *ass1*, *cad* and *sept6* were downregulated in *eif2b3^−/−^* zebrafish, especially in the MHB region where *lonp1* and *slc1a4* genes are up-regulated (Figure 4B). The MHB is a secondary organizer region that develops at the junction of the midbrain and hindbrain. Although the lens size was reduced, the mRNA expression of *crybb1* was not significantly changed in the *eif2b3* mutant (Figure S4).

## 3. Discussion

VWM disease is an autosomal recessive genetic leukodystrophy associated with mutation in one of the five subunits of *EIF2B* as *EIF2B1-5* in humans [16]. In previous studies, a relationship with VWM was reported, due to the mutation of each subunit. *EIF2B2* mutations were found to cause complex instability by interfering with holocomplex formation, and *EIF2B5* mutations are responsible for a decrease in the functional units present in the cells of VWM disease patients [17,18]. In order to understand the pathological mechanisms of each mutation, an animal model reproducing the phenotype by induced mutations was developed. Models due to point mutations of *Eif2b4* and *Eif2b5* in mice were reported, which expressed the characteristic phenotype of VWM disease [9,19,20].

Previous studies have undertaken proteomic profiling of the VWM disease model, based on the *Eif2b5* mutant model [6,7,8]. In astrocytes of WT and *Eif2b5^ho^* mice, 80 proteins were changed, 50% of which were related to secretory pathways [6]. Protein profiling of embryonic fibroblasts (MEFs) isolated from *Eif2b5^R132H/R132H^* mice revealed unbalanced stoichiometry of proteins involved in oxidative phosphorylation and the components of the mitochondrial translation machinery [7]. Changes in brain proteins in *Eif2b5^R132H/R132H^* mice showed that dysregulation of mitochondrial functions, altered proteasomal activity, and impaired balance between protein synthesis and degradation play a role in VWM pathology [8]. These results show that the disease characteristics of VWM disease are closely related to changes in proteins in the *Eif2b* mutant model.

In the present study, we identified four significanlty increased proteins DEPs in *eif2b3^−/−^* larvae: the neutral amino acid transporter SLC1A4 (*slc1a4*), Lon protease (*lonp1*), 4-aminobutyrate aminotransferase (*abat*), and probable 2-oxoglutarate dehydrogenase E1 component DHKTD1 (*dhtkd1*) based on comparative proteomics analysis. The DHKTD1 was increased in the proteome results, however, it was not changed at the mRNA level. Understanding the effect of upregulation of proteins in the *eif2b3* mutant will lead to increased understanding of the pathology of VWM disease.

The increase of the neutral amino acid transporter SLC1A4 (the alanine, serine, cysteine, and threonine transport, ASCT1) in *eif2b3^−/−^* larvae was also validated using WISH (Figure 4A). The neutral amino acid transporter SLC1A4 is a major *d*-serine uptake system in astrocytes, and contributes significantly to the uptake of *l*-serine in primary neurons [21]. SLC1A4 appears to be regulated to meet metabolic demands by differentiating and mature neurons through the transport of glial- and blood-borne small neutral amino acids [22]. The mis-regulation as *SCL1A4* mutation in the brain can change the levels of neutral amino acids like *l*-serine, *d*-serine, *l*-alanine, *l*-threonine, and glycine, which can affect brain functions in ways such as decreased volume of different brain regions, altered gene expression, and motor and learning deficits [23]. *l*-Serine plays an essential role in neuronal development and function. *l*-Serine biosynthesis is largely confined to astrocytes, and it is shuttled to the neuronal cells by SLC1A4. SLC1A4 was revealed as physiological regulator of *d*-serine metabolism, a coagonist of NMDA receptors [23].

Recently, recessive mutations in *SLC1A4* gene have been associated with hypomyelination, developmental delay, and microcephaly [24]. *SLC1A4* mutations were identified in two affected siblings with severe intellectual disability (ID), microcephaly and spasticity, from an Ashkenazi Jewish consanguineous family [25]. Additional SLC1A4 deficiency was described in patients with neurodevelopmental disorders, presenting with thin corpus callosum, in addition to the phenotypes of developmental delay, microcephaly, and seizures [26,27,28]. Glutamate excitotoxicity is associated with neurodegenerative disorders, including demyelination. As one of the toxic demyelination models, cuprizone induces demyelination and axonal damage. *Slc1a4* was upregulated in cuprizone-induced demyelination, especially in the corpus callosum in a region-specific manner [29]. These results suggest that the failure of proper *SLC1A4* expression may be in part the consequence of astroglial dysfunction, glutamate signaling, demyelination, axonal damage, and thin corpus callosum, related to demyelinating neurological diseases.

Lon protease is a mitochondrial matrix protease, crucial for the maintenance of mitochondrial homeostasis, and Lon dysregulation is involved in cancer and in the cerebral, ocular, dental, auricular and skeletal syndrome [30]. In the VWM disease model, *Eif2b* mutant, functional abnormalities of mitochondria can be characterized, comprising the abnormal mitochondrial content phenotype of the mutant cells [7,15,31]. The increase in Lon protease was verified in the zebrafish model through the WISH method (Figure 4A). The increased Lon protease in *eif2b3^−/−^* may be an important factor in the malfunction of mitochondria in VWM disease. Similarly, abnormal upregulation compared to WT in *eif2b3^−/−^* may be associated with pathological symptoms in the brain in VWM disease. In this study, new clues that can interpret the mechanism of pathology of VWM, by identifying the proteins changed in the mutant model, and their relevance needs to be clarified through further studies.

When a specific gene is reduced in the mutant model, its protein is likely to be a one whose expression is directly affected. In our study, crystallin proteins accounted for most of the reduced proteins which are annotated to structural constituents of the eye lens and visual perception categories in DAVID analysis with Opsin-1 (Figure 3B and Table 1). Although it was reported that α-crystalline B chains were significantly increased in astrocytes derived from VWM disease models in previous studies, the type of crystalline (β and γ, not α), were significantly decreased in *eif2b3^−/−^* [11,32]. γ-crystallin is particularly rich in the core region of the lens of the human eye [33]. From a genome-wide polysome-profiling strategy, it was found that a cohort of lens-associated crystallin isoform mRNAs is under the control of a specific mechanism of translation [34]. The optic atrophy, followed by blindness, in one of representative phenotypes in VWM disease patients, are possibly due to a significant decrease in β- and γ-crystalline, which plays an important role in the visual system.

Among the downregulated proteins, citrulline-aspartate ligase was reduced in mutant larvae as confirmed by WISH (Figure 4B). Citrulline-aspartate ligase in zebrafish has homology with the arginosuccinate synthase (ASS1) protein in humans (Table 1). Arginosuccinate synthase 1 is one of the enzymes of the urea cycle, and it catalyzes the formation of arginosuccinate from aspartate, citrulline and ATP and together with arginosuccinate lyase, and it is responsible for the biosynthesis of arginine in most body tissues. When arginosuccinate synthase 1 is deficient, the urea cycle does not work, causing hyperammonemia, in which the concentration of ammonia increases in blood. Ammonia is a neurotoxin involved in the pathogenesis of neurological conditions associated with hyperammonemia, including hepatic encephalopathy and brain edema [35,36]. Patients with neonatal-onset hyperammonemia should have early therapy to avoid severe brain damage [37]. Further studies should be conducted into the effect of the *eif2b3^−/−^* on the reduction of arginosuccinate synthase 1, but arginosuccinate synthase 1 may be suggested as a mechanism for the phenotype in the brain of VWM.

Using WISH analysis, we found a specific brain region in which both upregulation (*lonp1* and *slc1a4*) and downregulation (*ass1* and *cad*) of genes occurs. The MHB, also called the isthmic organizer, is a secondary organizer region that develops at the junction of the midbrain and hindbrain [38]. The MHB expresses signaling molecules that regulate the differentiation and patterning of the adjacent neuroepithelium. This organization allows for the development of the midbrain and hindbrain, as well as the specification of neuronal subtypes in these regions. Further studies focusing on the differential expression of these genes in MHB and their possible role in the pathogenesis of VWM disease, will be needed.

In conclusion, this study proposed candidate proteins as key regulators of the progression of VWN disease. We used quantitative proteomic analysis in the first EIF2B3 animal model of VWN disease. Abnormalities of myelin gene expression and glial cell differentiation were observed in the *eif2b3* knockout zebrafish, and the DEPs were profiled by comparative quantitative proteomic analysis. Proteins related to the etiological phenomenon of VWM were identified, and the expression patterns were verified, thereby providing information on the molecular mechanisms underlying the symptoms of VWN disease.

## 4. Materials and Methods

### 4.1. Zebrafish Husbandry

Animal experiments were conducted according to approved guidelines and regulations of the Institutional Animal Care and Use Committee at the Animal Ethics Committee of Chungnam National University (Approval number: 202012A-CNU-170). Adult fish were reared under standard conditions with a 14 h/10 h light/dark cycle. We obtained embryos by natural mating, and zebrafish were reared in egg water at 28.5 °C. WT zebrafish were obtained from the Zebrafish Center for Disease Modeling.

### 4.2. Maintenance of eif2b3 Knockout Zebrafish

To understand the in vivo role of EIF2B3, we established a zebrafish knockout model utilizing the CRISPR/Cas9 system, as described previously [39]. PCR primers for genotyping of *eif2b3^−/−^* zebrafish were forward primer, 5′-TGTTCGGGATGGAGCTACAG-3′, and reverse primer, 5′ -TTTGTTGCCCACAGGAAGCA-3′. PCR products (20 μL) were re-annealed in a thermal cycler under the following conditions: 95 °C for 2 min, 95–85 °C at 2 °C/s, 85–25 °C at 0.1 °C/s, then kept at 4 °C.

### 4.3. Protein Sample Preparation

The deyolked zebrafish embryos from each group were lysed with ice-cold radioimmunoprecipitation assay buffers (Thermo Fisher Scientific, Waltham, MA, USA) containing protease inhibitors (Thermo Fisher Scientific) using a homogenizer. Sonication was applied directly to all samples for 2 min (output 30%, 5 sec on and off intervals) on ice and centrifuged at 4 °C at 16,000× *g* for 10 min [14]. The soluble fractions were moved into new sample tubes, and the protein concentration measured using BCA kits (Thermo Fisher Scientific). We placed 100 μg of the protein samples (triplicates) from each group (15 embryos) to new sample tubes and added 5 mM of dithiothreitol (Sigma-Aldrich, St. Louis, MO, USA) and incubated at 56 °C for 30 min, followed by treatment with 15 mM iodoacetamide (Sigma-Aldrich) in the dark for 30 min. Then, ice-cold acetone was added slowly to the samples for protein precipitation and kept for 18 hr in a −20 °C freezer. The samples were centrifuged at 4 °C at 16,000× *g* for 10 min and supernatants were removed. The protein pellets were dissolved in 50 mM tetraethylammonium bromide (Sigma-Aldrich) and the protein concentration measured again. The pH was adjusted to 8 and trypsin (1:50) was directly added to the sample and left to digest for 18 hr at 37 °C. Finally, 1% trifluoroacetic acid (Sigma-Aldrich) added to complete the trypsin digestion. The peptides were dried in speed vac dryer at low temperature and directly dissolved in 50 mM tetraethylammonium bromide for 2-plex tandem mass tag (TMT) labeling (Thermo Fisher Scientific). After checking the peptide concentration using a Pierce™ Quantitative Colorimetric Peptide Assay Kit (Thermo Fisher Scientific), equal amounts of peptides from WT and *eif2b3^−/−^* group were labeled (*n* = 3) and placed in one sample tube. The pooled peptide samples were fractionated using a Pierce™ High-pH Reversed-Phase Peptide Fractionation Kit (Thermo Fisher Scientific).

### 4.4. Comparative Proteome Analysis

The fractionated samples were dissolved in 10 μL of 2% in 0.1% formic acid solution, and 500 ng of each fraction was loaded onto a nano-LC 1D plus system (Eksigent, Framingham, MA, USA) consisting of an in-house C_18_ resin (5 µm Proteo 100 Å; Phenomenex Inc., Torrance, CA, USA) and a capillary column (ID 75 µm, OD 150 µm; Molex, Lisle, IL, USA). Elution was conducted using a gradient liquid chromatography method (5–25% acetonitrile for 90 min) and analyzed with an LTQ-Orbitrap Velos mass spectrometer (Thermo Fisher Scientific) in positive ion mode at the Mass Spectrometry Convergence Research Center. The *m*/*z* data collection range was set at *m*/*z* 300–1800, and higher-energy collisional dissociation collision mode was used for fragmentation. The mass spectrometry proteomics data have been deposited to the ProteomeXchange Consortium via the PRIDE partner repository with the dataset identifier PXD023933.

### 4.5. Bioinformatics

All mass spectra data were input to MaxQuant 1.5.1.0 to obtain bioinformatics information, and the zebrafish proteome database (updated 25 October 2018) was downloaded from UniProt (https://www.uniprot.org/proteomes/UP000000437, accessed on 4 March 2021). All MS/MS spectra obtained were analyzed using MaxQuant software (https://www.maxquant.org/, accessed on 4 March 2021), allowing a maximum false-discovery rate of 1% for the protein and peptides. GO, InterPro, and KEGG pathways were analyzed using DAVID Functional Annotation Bioinformatics Microarray Analysis (https://david.ncifcrf.gov/, accessed on 4 March 2021). Perseus 1.6.0.7 was used for clustering protein groups depending on the protein regulation patterns. The STRING analytical tool (https://string-db.org/, accessed on 4 March 2021) was used to search specific protein networks according to regulation differences resulting from mutation of *eif2b3*.

### 4.6. Whole-Mount In Situ Hybridization

Whole-mount in situ hybridization (WISH) was performed essentially as described previously [13]. WISH was performed using probes for *mbp*, *olig2*, *nestin*, *lonp1*, *slc1a4*, *abat*, *ass1*, *cad*, *sept6*, *dhtkd1*, *psat1*, *xbp1*, *atf4*, *atf6*, and *crybb* (Table S2). Briefly, staged embryos were fixed overnight in 4% PFA, then dehydrated in a methanol gradient. Embryos were then rehydrated in phosphate buffered saline containing 0.1% Tween-20 (PBST). Embryos were permeabilized by proteinase K digestion and then hybridized with digoxin-labeled probes overnight at 70 °C. The next day, embryos were washed in a preheated mixture of 50% saline sodium citrate containing 0.1% Tween-20 and 50% hybridization solution at 70 °C. Embryos were washed again at room temperature and incubated in staining solution in the dark until sufficient staining appeared. Embryos were mounted in glycerol and visualized using a Nikon AZ100 microscope (Nikon). Images were captured using a Nikon DIGITAL SIGHT DS-Fil1 digital camera (Nikon) and processed with NIS-Elements F 3.0 (Nikon).

## Figures and Tables

**Table 1:** Differentially expressed proteins.

Protein Level	*D. rerio*				Human Homology		
	Protein IDs	Protein Names	Gene Names	Ratio (KO vs. WT)	Protein IDs	Protein Names	Gene Names
*Up*	A0A0R4IH79	Lon protease homolog, mitochondrial	*lonp1*	2.97	P36776	Lon protease homolog, mitochondrial	*LONP1*
*Up*	Q5PRA2	Probable 2-oxoglutarate dehydrogenase E1 component DHKTD1, mitochondrial	*dhtkd1*	2.21	Q96HY7	Probable 2-oxoglutarate dehydrogenase E1 component DHKTD1, mitochondrial	*DHTKD1*
*Up*	I3IRW7	4-aminobutyrate aminotransferase	*abat*	2.09	P80404	4-aminobutyrate aminotransferase, mitochondrial	*ABAT*
*Up*	D7RVS1	Neutral amino acid transporter SLC1A4	*slc1a4*	2.73	P43007	Neutral amino acid transporter A	*SLC1A4*
*Down*	B6IDE1	Slow myosin heavy chain 2	*smyhc2*	0.37	P12883	Myosin-7	*MYH7*
*Down*	F1R3Q3	Septin 6	*sept6*	0.46	Q14141	Septin-6	*SEPTIN6*
*Down*	A7E2K7	Crystallin, gamma M2d12	*crygm2d12*	0.37	P07316	Gamma-crystallin B	*CRYGB*
*Down*	Q6DH12	Crystallin, gamma M2d13	*crygm2d13*	0.36	P07316	Gamma-crystallin B	*CRYGB*
*Down*	Q9W6A9	Opsin-1, short-wave-sensitive 1	*opn1sw1*	0.20	P03999	Short-wave-sensitive opsin 1	*OPN1SW*
*Down*	Q15I84	Crystallin gamma EM2-5	*crygm2d5*	0.25	P07316	Gamma-crystallin B	*CRYGB*
*Down*	Q6DGX6	Crystallin gamma EM2-8	*crygm2d8*	0.20	P07316	Gamma-crystallin B	*CRYGB*
*Down*	Q45FX9	BetaA2-2-crystallin	*cryba2b*	0.47	P53672	Beta-crystallin A2	*CRYBA2*
*Down*	A0A0R4IYV7	Citrulline--aspartate ligase	*N/A*	0.19	P00966	Argininosuccinate synthase	*ASS1*
*Down*	Q52JI6	Beta A1-2-crystallin	*cryba1l1*	0.46	P05813	Beta-crystallin A3	*CRYBA1*
*Down*	Q6DGY4	Crystallin, beta A1b	*cryba1b*	0.44	P05813	Beta-crystallin A3	*CRYBA1*
*Down*	U3JAV8	Aspartate carbamoyltransferase	*cad*	0.46	P27708	CAD protein	*CAD*
*Down*	Q5XTN0	Crygmx protein	*crygmx*	0.37	P07316	Gamma-crystallin B	*CRYGB*
*Down*	B0S6M3	Crystallin, gamma M2d1	*crygm2d1*	0.25	P07316	Gamma-crystallin B	*CRYGB*
*Down*	Q52JI7	Beta A4-crystallin	*cryba4*	0.45	P53673	Beta-crystallin A4	*CRYBA4*
*Down*	A8E4S8	Crystallin, gamma M2d10	*crygm2d10*	0.25	P07316	Gamma-crystallin B	*CRYGB*
*Down*	Q6DGY7	Gamma-crystallin N-B	*crygnb*	0.47	Q8WXF5	Gamma-crystallin N	*CRYGN*
*Down*	Q6IQU2	Beta A2-crystallin	*cryba2a*	0.43	P53672	Beta-crystallin A2	*CRYBA2*
*Down*	B0V191	Crystallin, gamma M2d18	*crygm2d18*	0.23	P07316	Gamma-crystallin B	*CRYGB*
*Down*	E7F8M1	Crystallin, beta B1,-like 1	*crybb1l1*	0.44	P53674	Beta-crystallin B1	*CRYBB1*
*Down*	Q90WT1	Beta B1-crystallin	*crybb1*	0.43	P53674	Beta-crystallin B1	*CRYBB1*

N/A; not available.

## Data Availability

Data are available via ProteomeXchange with identifier PXD023933.

## Supplementary Materials

Supplementary Materials can be found at https://www.mdpi.com/1422-0067/22/5/2707/s1. Figure S1: Sodium dodecyl sulfate-polyacrylamide gel electrophoresis after yolk protein remove, Figure S2: Whole-mount in situ hybridization of WT and *eif2b3^−/−^* probed for *dhtkd1*, *past1*, and *xbp1*, Figure S3: Whole-mount in situ hybridization of WT and *eif2b3^−/−^* probed for *atf4* and *atf6*. Figure S4: Whole-mount in situ hybridization of WT and *eif2b3^−/−^* probed for *crybb1*, Table S1: All detected protein lists. Table S2: Primer sequences used for in situ probe synthesis. Hybridization temperature for each probe was 70 °C.

## References

[1] Abbink T.E.M., Wisse L.E., Jaku E., Thiecke M.J., Voltolini-Gonzalez D., Fritsen H., Bobeldijk S., Ter Braak T.J., Polder E., Postma N.L. (2019). Vanishing white matter: Deregulated integrated stress response as therapy target. Ann. Clin. Transl. Neurol..

[2] Van der Knaap M.S., Fogli A., Boespflug-Tanguy O., Abbink T.E.M., Schiffmann R., Adam M.P., Ardinger H.H., Pagon R.A., Wallace S.E., Bean L.J.H., Stephens K., Amemiya A. (1993). Childhood Ataxia with Central Nervous System Hypomyelination/Vanishing White Matter. GeneReviews((R)).

[3] Van der Knaap M.S., Pronk J.C., Scheper G.C. (2006). Vanishing white matter disease. Lancet Neurol..

[4] Yavuz H. (2017). A Review of Infantile Vanishing White Matter Disease and A New Mutation. Acta Neurol. Taiwan.

[5] Van der Lei H.D., van Berkel C.G., van Wieringen W.N., Brenner C., Feigenbaum A., Mercimek-Mahmutoglu S., Philippart M., Tatli B., Wassmer E., Scheper G.C. (2010). Genotype-phenotype correlation in vanishing white matter disease. Neurology.

[6] Wisse L.E., Penning R., Zaal E.A., van Berkel C.G.M., Ter Braak T.J., Polder E., Kenney J.W., Proud C.G., Berkers C.R., Altelaar M.A.F. (2017). Proteomic and Metabolomic Analyses of Vanishing White Matter Mouse Astrocytes Reveal Deregulation of ER Functions. Front. Cell. Neurosci..

[7] Raini G., Sharet R., Herrero M., Atzmon A., Shenoy A., Geiger T., Elroy-Stein O. (2017). Mutant eIF2B leads to impaired mitochondrial oxidative phosphorylation in vanishing white matter disease. J. Neurochem..

[8] Gat-Viks I., Geiger T., Barbi M., Raini G., Elroy-Stein O. (2015). Proteomics-level analysis of myelin formation and regeneration in a mouse model for Vanishing White Matter disease. J. Neurochem..

[9] Geva M., Cabilly Y., Assaf Y., Mindroul N., Marom L., Raini G., Pinchasi D., Elroy-Stein O. (2010). A mouse model for eukaryotic translation initiation factor 2B-leucodystrophy reveals abnormal development of brain white matter. Brain.

[10] Leferink P.S., Dooves S., Hillen A.E.J., Watanabe K., Jacobs G., Gasparotto L., Cornelissen-Steijger P., van der Knaap M.S., Heine V.M. (2019). Astrocyte Subtype Vulnerability in Stem Cell Models of Vanishing White Matter. Ann. Neurol..

[11] Zhou L., Li P., Chen N., Dai L.F., Gao K., Liu Y.N., Shen L., Wang J.M., Jiang Y.W., Wu Y. (2019). Modeling vanishing white matter disease with patient-derived induced pluripotent stem cells reveals astrocytic dysfunction. CNS Neurosci. Ther..

[12] Keefe M.D., Soderholm H.E., Shih H.Y., Stevenson T.J., Glaittli K.A., Bowles D.M., Scholl E., Colby S., Merchant S., Hsu E.W. (2020). Vanishing white matter disease expression of truncated EIF2B5 activates induced stress response. Elife.

[13] Lee Y.R., Kim S.H., Ben-Mahmoud A., Kim O.H., Choi T.I., Lee K.H., Ku B., Eum J., Kee Y., Lee S. (2021). Eif2b3 mutants recapitulate phenotypes of Vanishing White Matter Disease and validate novel disease alleles in zebrafish. Hum. Mol. Genet..

[14] Link V., Shevchenko A., Heisenberg C.P. (2006). Proteomics of early zebrafish embryos. BMC Dev. Biol..

[15] Herrero M., Mandelboum S., Elroy-Stein O. (2019). eIF2B Mutations Cause Mitochondrial Malfunction in Oligodendrocytes. Neuromol. Med..

[16] Leegwater P.A., Vermeulen G., Konst A.A., Naidu S., Mulders J., Visser A., Kersbergen P., Mobach D., Fonds D., van Berkel C.G. (2001). Subunits of the translation initiation factor eIF2B are mutant in leukoencephalopathy with vanishing white matter. Nat. Genet..

[17] Alsalem A., Shaheen R., Alkuraya F.S. (2012). Vanishing white matter disease caused by EIF2B2 mutation with the presentation of an adrenoleukodystrophy phenotype. Gene.

[18] Mierzewska H., van der Knaap M.S., Scheper G.C., Jurkiewicz E., Schmidt-Sidor B., Szymanska K. (2006). Leukoencephalopathy with vanishing white matter due to homozygous EIF2B2 gene mutation. First Polish cases. Folia Neuropathol..

[19] Dooves S., Bugiani M., Postma N.L., Polder E., Land N., Horan S.T., van Deijk A.L., van de Kreeke A., Jacobs G., Vuong C. (2016). Astrocytes are central in the pathomechanisms of vanishing white matter. J. Clin. Investig..

[20] Terumitsu-Tsujita M., Kitaura H., Miura I., Kiyama Y., Goto F., Muraki Y., Ominato S., Hara N., Simankova A., Bizen N. (2020). Glial pathology in a novel spontaneous mutant mouse of the Eif2b5 gene: A vanishing white matter disease model. J. Neurochem..

[21] Yamamoto T., Nishizaki I., Nukada T., Kamegaya E., Furuya S., Hirabayashi Y., Ikeda K., Hata H., Kobayashi H., Sora I. (2004). Functional identification of ASCT1 neutral amino acid transporter as the predominant system for the uptake of L-serine in rat neurons in primary culture. Neurosci. Res..

[22] Sakai K., Shimizu H., Koike T., Furuya S., Watanabe M. (2003). Neutral amino acid transporter ASCT1 is preferentially expressed in L-Ser-synthetic/storing glial cells in the mouse brain with transient expression in developing capillaries. J. Neurosci..

[23] Kaplan E., Zubedat S., Radzishevsky I., Valenta A.C., Rechnitz O., Sason H., Sajrawi C., Bodner O., Konno K., Esaki K. (2018). ASCT1 (Slc1a4) transporter is a physiologic regulator of brain d-serine and neurodevelopment. Proc. Natl. Acad. Sci. USA.

[24] Damseh N., Simonin A., Jalas C., Picoraro J.A., Shaag A., Cho M.T., Yaacov B., Neidich J., Al-Ashhab M., Juusola J. (2015). Mutations in SLC1A4, encoding the brain serine transporter, are associated with developmental delay, microcephaly and hypomyelination. J. Med. Genet..

[25] Srour M., Hamdan F.F., Gan-Or Z., Labuda D., Nassif C., Oskoui M., Gana-Weisz M., Orr-Urtreger A., Rouleau G.A., Michaud J.L. (2015). A homozygous mutation in SLC1A4 in siblings with severe intellectual disability and microcephaly. Clin. Genet..

[26] Heimer G., Marek-Yagel D., Eyal E., Barel O., Oz Levi D., Hoffmann C., Ruzzo E.K., Ganelin-Cohen E., Lancet D., Pras E. (2015). SLC1A4 mutations cause a novel disorder of intellectual disability, progressive microcephaly, spasticity and thin corpus callosum. Clin. Genet..

[27] Conroy J., Allen N.M., Gorman K., O’Halloran E., Shahwan A., Lynch B., Lynch S.A., Ennis S., King M.D. (2016). Novel European SLC1A4 variant: Infantile spasms and population ancestry analysis. J. Hum. Genet..

[28] Abdelrahman H.A., Al-Shamsi A., John A., Ali B.R., Al-Gazali L. (2019). A Novel SLC1A4 Mutation (p.Y191*) Causes Spastic Tetraplegia, Thin Corpus Callosum, and Progressive Microcephaly (SPATCCM) With Seizure Disorder. Child Neurol. Open.

[29] Azami Tameh A., Clarner T., Beyer C., Atlasi M.A., Hassanzadeh G., Naderian H. (2013). Regional regulation of glutamate signaling during cuprizone-induced demyelination in the brain. Ann. Anat..

[30] Pinti M., Gibellini L., Nasi M., De Biasi S., Bortolotti C.A., Iannone A., Cossarizza A. (2016). Emerging role of Lon protease as a master regulator of mitochondrial functions. Biochim. Biophys. Acta.

[31] Atzmon A., Herrero M., Sharet-Eshed R., Gilad Y., Senderowitz H., Elroy-Stein O. (2018). Drug Screening Identifies Sigma-1-Receptor as a Target for the Therapy of VWM Leukodystrophy. Front. Mol. Neurosci..

[32] Bugiani M., Boor I., van Kollenburg B., Postma N., Polder E., van Berkel C., van Kesteren R.E., Windrem M.S., Hol E.M., Scheper G.C. (2011). Defective glial maturation in vanishing white matter disease. J. Neuropathol. Exp. Neurol..

[33] Vendra V.P., Khan I., Chandani S., Muniyandi A., Balasubramanian D. (2016). Gamma crystallins of the human eye lens. Biochim. Biophys. Acta.

[34] Choudhuri A., Maitra U., Evans T. (2013). Translation initiation factor eIF3h targets specific transcripts to polysomes during embryogenesis. Proc. Natl. Acad. Sci. USA.

[35] Skowronska M., Albrecht J. (2012). Alterations of blood brain barrier function in hyperammonemia: An overview. Neurotox Res..

[36] Manakkat Vijay G.K., Hu C., Peng J., Garcia-Martinez I., Hoque R., Verghis R.M., Ma Y., Mehal W.Z., Shawcross D.L., Wen L. (2019). Ammonia-Induced Brain Edema Requires Macrophage and T Cell Expression of Toll-Like Receptor 9. Cell Mol. Gastroenterol. Hepatol..

[37] Batshaw M.L. (1984). Hyperammonemia. Curr. Probl. Pediatr..

[38] Wurst W., Bally-Cuif L. (2001). Neural plate patterning: Upstream and downstream of the isthmic organizer. Nat. Rev. Neurosci..

[39] Lee Y.R., Khan K., Armfield-Uhas K., Srikanth S., Thompson N.A., Pardo M., Yu L., Norris J.W., Peng Y., Gripp K.W. (2020). Mutations in FAM50A suggest that Armfield XLID syndrome is a spliceosomopathy. Nat. Commun..

