# Supplementary material for: Comparative Proteome Research in a Zebrafish Model for Vanishing White Matter Disease
Source: Int J Mol Sci. 2021 Mar 8;22(5):2707. doi: 10.3390/ijms22052707 (PMC7962458; doi:10.3390/ijms22052707)
Supplement: Supplementary file 1 [file ijms-22-02707-s001.pdf]

Supporting information

Supplemental Table 1. All detected protein lists.

Supplemental Table 2. Primer sequences used for in situ probe synthesis. Hybridization temperature for each probe was 70°C.

Gene	Primer	Nucleotide sequence (5' to 3')
nestin	forward	CCATGCAGCAAAGAGAAGAA
nestin	reverse	TAATACGACTCACTATAGGTGTGACTTGTAGACACAGAACTGC
lonp1	forward	TGGACGTCATTAACGAAGAGCTC
lonp1	reverse	TAATACGACTCACTATAGGGCCACTGACAATCCTGAACGCT
slc1a4	forward	ATGGTGCTTGTGTCCTGGAT
slc1a4	reverse	TAATACGACTCACTATAGGGCAGACCAATGGCTTCCAGAA
abat	forward	GCCACAACACATTCCAAGGCCA
abat	reverse	TAATACGACTCACTATAGGGAAGGGTCTCCCATCCACGTG
ass1	forward	TGGAACCAGTCATCGCTCCATGG
ass1	reverse	TAATACGACTCACTATAGGGTTGATCTTGCGCACCTCTCGGT
cad	forward	TGCTCCTAAGCGTGTGATCG
cad	reverse	TAATACGACTCACTATAGGGGAAATGCACCACAGACCCTC
sept6	forward	CCAACACTTATGAGCTGCAGGAG
sept6	reverse	TAATACGACTCACTATAGGGCTCAGTGCTTCCCACCACTGCAA
dhtkd1	forward	GGTGAGGCTAAATGGCTCCTGCA
dhtkd1	reverse	TAATACGACTCACTATAGGGCCTGTTGCAGAGCTTCTGTGG
psat1	forward	ATATTTGCAGGCGCGCAGAA
psat1	reverse	TAATACGACTCACTATAGGGTGCATTGTACAAAGACGCACG
xbp1	forward	CCATGGATACTCACAGCCCT
xbp1	reverse	TAATACGACTCACTATAGGGTTCTCGTAGCCGCCGAAAGAG
atf4	forward	TGAGCCTCTCTCCGTCTCACAT
atf4	reverse	TAATACGACTCACTATAGGGGGATGGATCTGGACCGACAG
atf6	forward	ACCAAAACTCGCCGCATGAGCAA
atf6	reverse	TAATACGACTCACTATAGGGGCCCACGAGGACTCCAACAG

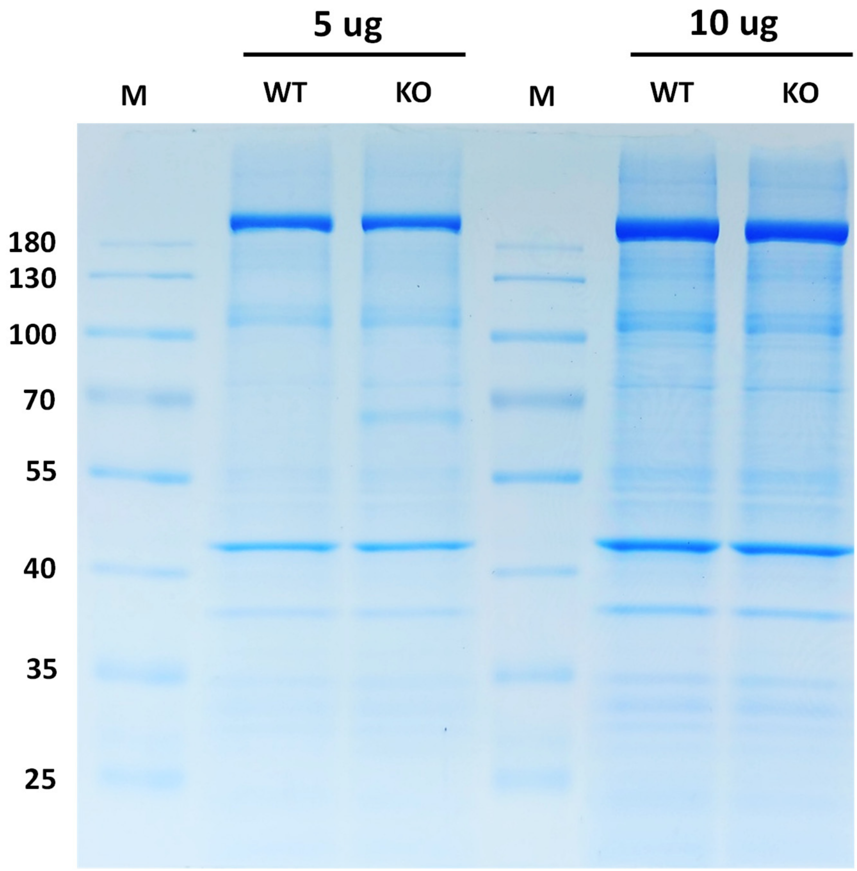

Figure S1. Sodium dodecyl sulfate-polyacrylamide gel electrophoresis after yolk protein removal.

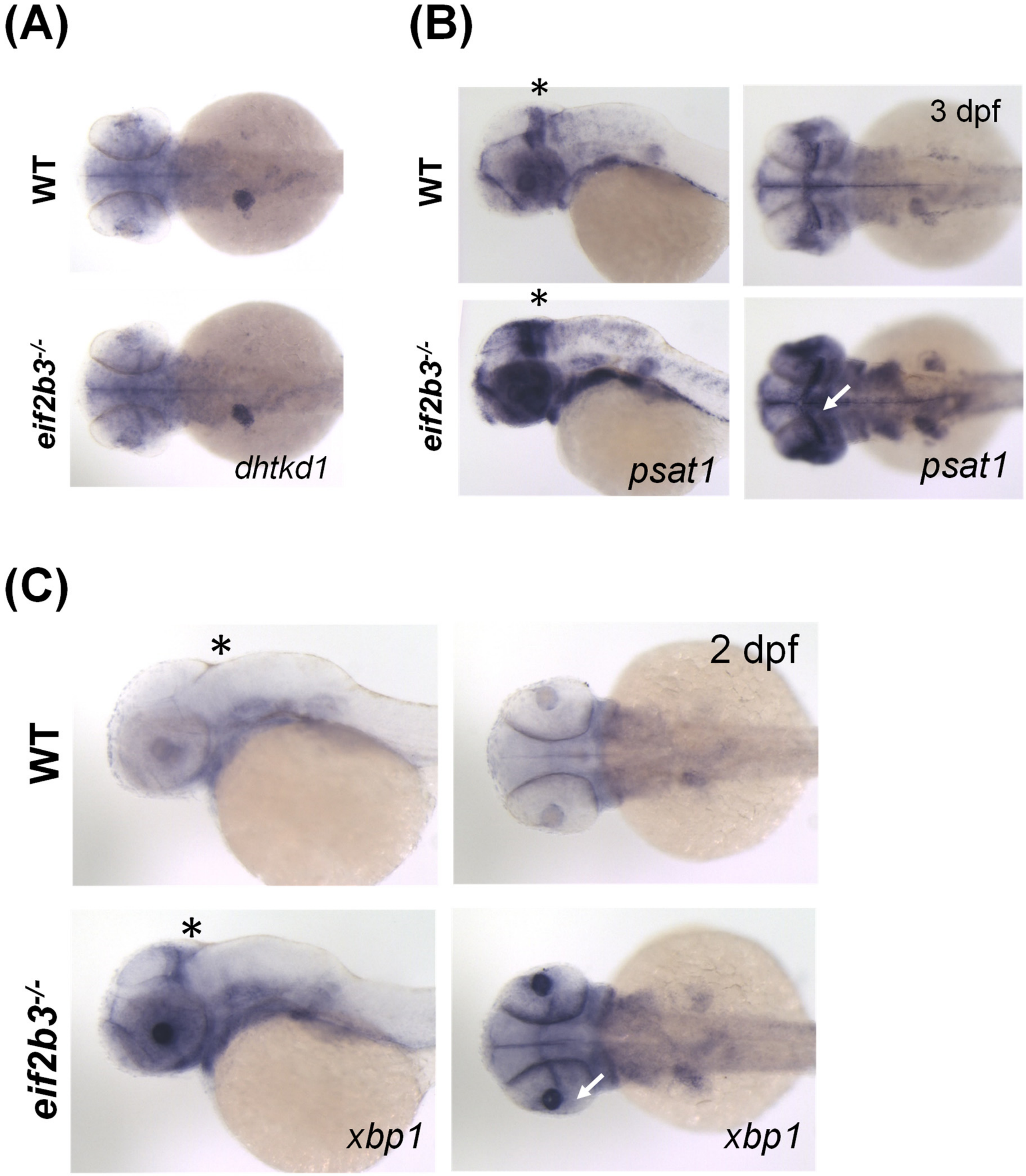

Figure S2. Whole-mount *in situ* hybridization of WT and *eif2b3*$^{-/-}$ probed for *dhtkd1, past1, and xbp1*.

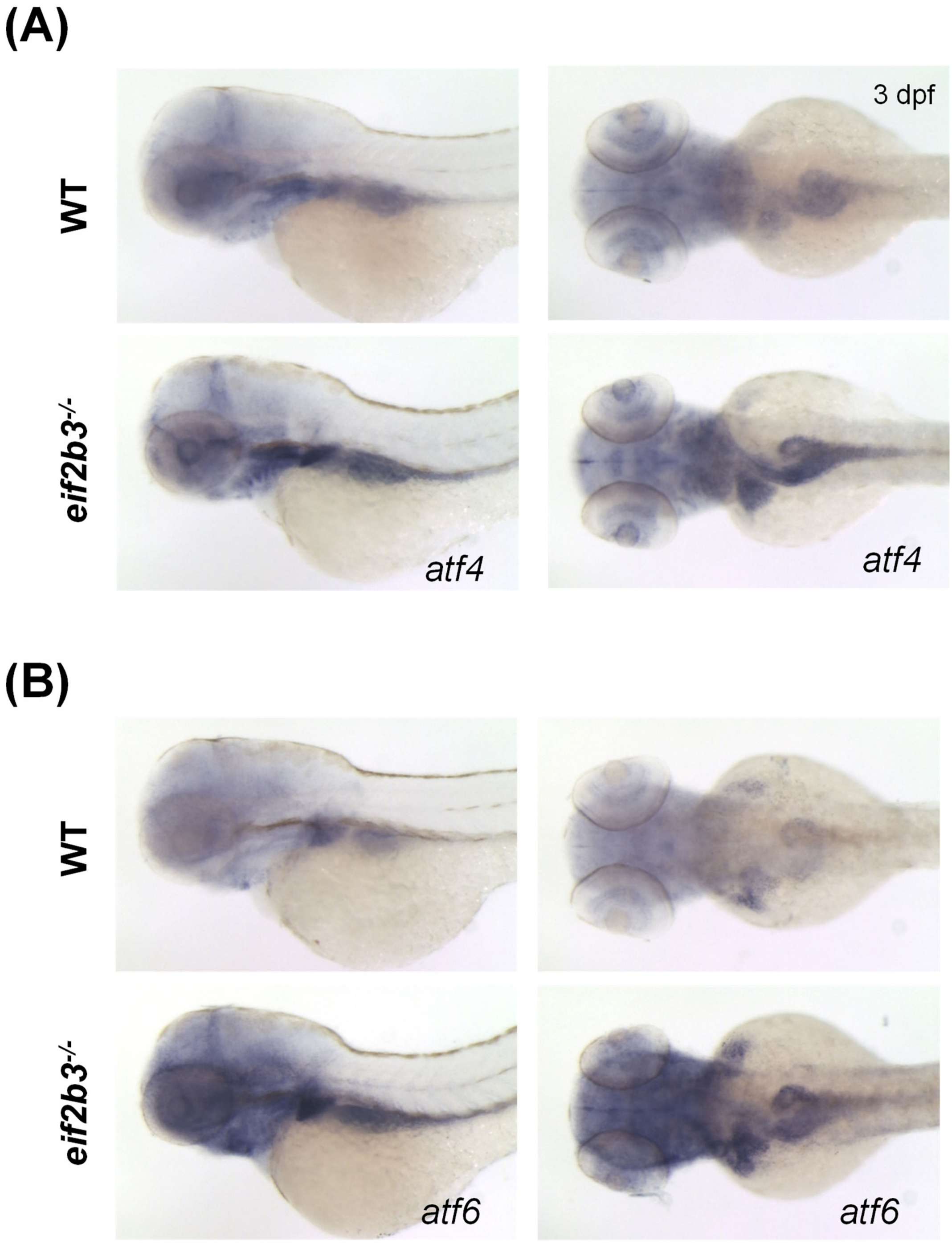

Figure S3. Whole-mount *in situ* hybridization of WT and *eif2b3*$^{-/-}$ probed for *atf4* and *atf6*.

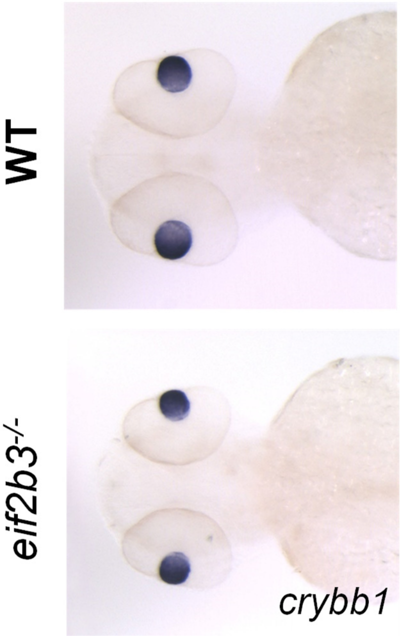

Figure S4 Whole-mount *in situ* hybridization of WT and *eif2b3*$^{-/-}$ probed for *crybb1*.
